# Action Video Gaming Experience Related to Altered Resting-State EEG Temporal and Spatial Complexity

**DOI:** 10.3389/fnhum.2021.640329

**Published:** 2021-06-29

**Authors:** Ruifang Cui, Jinliang Jiang, Lu Zeng, Lijun Jiang, Zeling Xia, Li Dong, Diankun Gong, Guojian Yan, Weiyi Ma, Dezhong Yao

**Affiliations:** ^1^MOE Key Lab for Neuroinformation, The Clinical Hospital of Chengdu Brain Science Institute, University of Electronic Science and Technology of China, Chengdu, China; ^2^School of Life Sciences and Technology, Center for Information in Medicine, University of Electronic Science and Technology of China, Chengdu, China; ^3^School of Human Environmental Sciences, University of Arkansas, Fayetteville, AR, United States

**Keywords:** action real-time strategy gaming, action video gaming, EEG microstate, multi-player online shooting gaming, omega complexity

## Abstract

Action video gaming (AVG) places sustained cognitive load on various behavioral systems, thus offering new insights into learning-related neural plasticity. This study aims to determine whether AVG experience is associated with resting-state electroencephalogram (rs-EEG) temporal and spatial complexity, and if so, whether this effect is observable across AVG subgenres. Two AVG games – League of Legends (LOL) and Player Unknown’s Battle Grounds (PUBG) that represent two major AVG subgenres – were examined. We compared rs-EEG microstate and omega complexity between LOL experts and non-experts (Experiment 1) and between PUBG experts and non-experts (Experiment 2). We found that the experts and non-experts had different rs-EEG activities in both experiments, thus revealing the adaptive effect of AVG experience on brain development. Furthermore, we also found certain subgenre-specific complexity changes, supporting the recent proposal that AVG should be categorized based on the gaming mechanics of a specific game rather than a generic genre designation.

## Introduction

Theories of learning science have reached a consensus that the brain changes physically, chemically, and functionally as a result of one’s life experience. This experience-dependent plasticity (Loevden et al., 2010) occurs when individuals are frequently exposed to a demanding task or a challenging environment that taxes certain cognitive systems of interest (Dale et al., 2020). Action video gaming (AVG) – a popular platform of entertainment – is cognitively demanding as it requires players to track multiple complex visual stimuli simultaneously, alert to the stimuli in peripheral vision, and make decisions and perform actions accordingly under time pressure (Green and Bavelier, 2003, 2012; Dale et al., 2020). It therefore offers important, new insights into the neural basis of learning.

The effect of AVG experience on behavioral and cognitive improvements has attracted increasing research attention over the past few decades (Dale et al., 2020). Although some studies have failed to observe an association between AVG experience and cognitive development (Owen et al., 2010; Boot et al., 2013), the majority of the literature has found a clear relationship – with a medium to large effect size – between AVG experience and the enhancement of various cognitive abilities (Wang et al., 2016; Bediou et al., 2018; Bavelier and Green, 2019). For example, AVG experience is related to the development of visual processing (Green and Bavelier, 2007), selective attention (Green and Bavelier, 2003), visuospatial processing (Green and Bavelier, 2006), visual working memory (Blacker et al., 2014), and visual-to-auditory attentional shifting (Franceschini et al., 2013). AVG experience may improve cognitive development – especially the abilities related to visual attention – because of AVG’s characteristic “action” elements, as AVG requires rapid responses to various types of stimuli under time pressure, a persistently high cognitive demand on divided attention, and timely shifts between focused and divided attentions (Bavelier and Green, 2019). This is also supported by interventional research showing that AVG training improved visual attentional performance (Green and Bavelier, 2003, 2006).

Unlike the earlier forms of AVG that are typically single-player sensorimotor tasks prioritizing only the action component (e.g., Super Mario Bros, Tetris, and Unreal Tournament 2004), the recent forms of AVG can be online, organized, multiplayer competitions demanding not only sensorimotor skills but also planning, strategizing, and cooperating with teammates (Gong et al., 2016; Dale and Green, 2017b, a). For example, League of Legends (LOL) – a multiplayer online battle arena video game that integrates AVG and real-time strategy (RTS) games – is a typical action real-time strategy gaming (ARSG) and is widely recognized as the world’s most iconic E-sport. Furthermore, the earlier forms of AVG (e.g., first or third person shooter games) typically use a linear storyline, thus offering different players a similar gaming experiences (Dale and Green, 2017b). By contrast, the recent forms of AVG gives each player a unique gaming experience because the storyline is produced by real-time interactions among multiple players who are playing the game at a given time. For example, the Player Unknown’s Battle Grounds (PUBG) game is a multi-player online shooting game (MOSG) that situates AVG into a large, complex game map, where up to one hundred players parachute onto an island and scavenge for weapons and equipment to defeat others with the last person or team alive winning the match. Thus, both LOL and PUBG are like traditional team sports (Kou and Gui, 2014; Mamun and Griffiths, 2019). Given the popularity of these new AVG subgenres, an examination of the effect of the new AVG subgenres on cognitive development allows us to lay important theoretic framework for the design and validation of the next-generation gaming-based therapy tools for cognitive deficits, such as cognitive decline (Toril et al., 2014), dyslexia (Franceschini et al., 2013), and amblyopia (Li et al., 2011).

Research has found an association between ARSG/RTS experience and enhanced cognitive functions. For example, ARSG/RTS experience is related to improved cognitive abilities, such as visual attention (Dale et al., 2019), speed of processing (Basak et al., 2008; Dale and Green, 2017a), visual perceptual learning (Kim et al., 2015), and cognitive flexibility (Glass et al., 2013). Furthermore, long-term ARSG/RTS experience may induce axon alterations that link the structures of the occipital-parietal loop and support visuospatial processing (Kowalczyk et al., 2018). Recently, research suggests that ARSG experience is related to enhancements of functional integration of insular subregions and the pertinent networks therein (Gong et al., 2015), functional integration between salience and central executive networks (Gong et al., 2016), and white matter networks in prefrontal networks, the limbic system, and sensorimotor networks (Gong et al., 2017). Cross-sectional research on LOL experts and non-experts also shows that ARSG experience is related to the enhanced visual attention (Qiu et al., 2018; Gan et al., 2020), theta spectrum power, beta spectrum power, network characteristics (Gong et al., 2019a), and visual working memory capacity (Yao et al., 2020). However, the effect of ARSG experience on the resting-state electroencephalogram (rs-EEG) temporal and spatial complexity still remains understudied.

The current study examined the relationship between ARSG/MOSG experience and cognitive development by comparing gaming experts and non-experts. This study differs from previous research in three ways. First, this study investigated rs-EEG temporal and spatial complexity, thus offering a new perspective on learning-related cognitive enhancements. Higher temporal and spatial complexity may guide individuals to opening channels into new regions of a complex multidimensional state space, thus further obtaining new possibilities for life (Jeffery and Rovelli, 2020). Second, this study used new data analysis methods and approaches to explore the improvements of large-scale networks. We conducted the EEG microstate analysis that can represent the spatial organization and temporal dynamics of large-scale cortical activities, as EEG offers high temporal resolution to indicate the temporal complexity (Gao et al., 2017; Michel and Koenig, 2018). In addition, we used the omega complexity analysis to assess the degree of synchronization among spatially large-scale brain regions, which can reflect the spatial complexity (Gao et al., 2017). Thus, this study can extend the previous rs-fMRI findings that brain improvements may occur at a global level as indicated by large-scale network data (Gong et al., 2015, 2016, 2017). Third, this study examined the recent forms of AVG (i.e., ARSG/MOSG) – the AVG genres that are becoming increasingly popular but still remain understudied. Finally, this study supports the recent proposal that AVG should be categorized based on the gaming mechanics of a specific game rather than a generic genre designation.

## Experiment 1

Experiment 1 examined the rs-EEG microstate and omega complexity in LOL experts and non-experts. LOL was used in this experiment for two reasons. First, LOL is one of the most popular ARSG subgenre, as a recent survey showed that LOL was played by over 67 million people per month, 27 million people per day, and over 7.5 million people concurrently during peak hours^1^. Second, research has consistently shown that LOL experts have enhanced visual attention (Qiu et al., 2018; Gan et al., 2020), theta spectrum power, beta spectrum power, network characteristics (Gong et al., 2019a), visual working memory capacity (Yao et al., 2020), functional integration (Gong et al., 2015, 2016), and structural connections (Gong et al., 2017).

### Participants

Experiment 1 used the recruitment procedure established in previous research (Qiu et al., 2018; Gong et al., 2019c; Gan et al., 2020). All participants in this study were college students of the University of Electronic Science and Technology of China (UESTC), who responded to the recruitment flyers posted on campus or on the Internet forums hosted by the UESTC. Prior to this experiment, participants completed a survey of demographic information, including age, sex, vision, handedness, and history of mental and neurological diseases. All participants were male, right-handed and had a normal or corrected-to-normal vision and had no history of mental and neurological diseases. Participants also completed a Self-Rating Depression Scale (SDS), a Self-Rating Anxiety Scale (SAS), and a seven-question Game Addiction Questionnaire. Based on the results, 6 additional individuals were excluded from the final sample, because they had moderate/severe depression or anxiety (i.e., SDS ≥ 63 or SAS ≥ 60) (*n* = 4), or Internet gaming disorders (*n* = 2). The participants also reported (1) their LOL gaming experience over the immediate recent 2 years and their current Expertise Gaming Ranking of LOL, (2) their LOL ID, which was used to verify their self-reported LOL gaming experience and expertise, as the LOL Expertise Ranking is also provided by the LOL game software, and (3) their video gaming experience of genres other than LOL over the immediate recent 2 years, which was used to ensure that LOL was the primary game genre for all participants, including both experts and non-experts, recruited in this experiment.

Following previous research (Qiu et al., 2018; Gong et al., 2019c; Gan et al., 2020), this study defined group membership using both time- and skill-based criteria. The experts had at least 2 years of LOL gaming experience and were LOL masters based on their Expertise Gaming Ranking provided by the LOL gaming software (the top 7% of players) – an objective, commonly used tool to quantify the relative gaming skill levels of LOL players. The non-experts had less than 0.5 years of LOL gaming experience and were LOL non-experts according to their Expertise Gaming Rankings (the lowest 29.92-45.11% of players) (Qiu et al., 2018; Gan et al., 2020). Only the individuals who were identified as either LOL experts or non-experts were invited to participate in this experiment. The participants were 22 LOL experts (*M* = 19.84 years, *SD* = 1.34) and 28 age-matched LOL non-experts (*M* = 20.90 years, *SD* = 3.26). Informed consent was obtained before the experiment, and the test was approved by the UESTC Ethics Board. To minimize participant bias, the participants were not informed of their group membership or the purpose of this study.

### EEG Recording and Preprocessing

Participants were tested individually in a recording room with electrical and sound shielding, where they were seated on a comfortable chair and were asked to remain relaxed and keep their eyes closed. Eye-closed rs-EEG signals were recorded for 5 min using an EEG32-BT EEG amplifier (BORUIEN, China), with the electrodes located according to the 10-20 system, and the signals digitized with a 1000-Hz sampling rate (Klem et al., 1999). All signals were amplified with a 0.05-100-Hz bandpass filter and online-referenced to the FCz. The impedance for all electrodes was kept below 5 KΩ.

The off-line EEG preprocessing were conducted using EEGLAB (Delorme and Makeig, 2004) in MATLAB 2013b (MathWorks, Natick, NA). EEG data were first re-referenced to “infinity” zero using the reference electrode standardization technique (REST) (Yao, 2001; Dong et al., 2017). The re-referenced data were then filtered by FIR-Butterworth filters, with half-power cutoffs of 0.10 Hz and 80 Hz (roll-off = 12 dB/oct). A 49–51 Hz notch-filter was also used to eliminate power frequency interferences. The bad channels were discarded by visual inspection and were interpolated using spherical method. Data portions contaminated by eye blinks and eye movements were corrected by independent component analysis (ICA). The ICA-corrected data then were segmented into 2s epochs and EEG epochs with amplitude values exceeding ± 100 μV at any electrode deleted (Luck, 2014). On an average, 141.95 (S.D. = 14.90) artifact-free EEG epochs were available for each expert, and 145.86 (S.D. = 13.83) epochs for each non-expert. The number of available epochs did not significantly different between groups (*p* = 0.33).

### EEG Microstate Analysis

According to the procedure established by previous research (Koenig et al., 1999), the EEG epochs were filtered using 2-20-Hz band-pass filter. Then, EEG microstate analyses were performed using the microstates 0.3 toolbox in MATLAB 2013b. Only the map topographies at peaks of global field power (GFP) were used for further analysis, because EEG map topographies remain stable around peaks of GFP that was EEG potential variance across all electrodes, and change around the troughs (Gao et al., 2017). Past research suggested that four widely reported canonical resting-state EEG microstate classes (i.e., class A, B, C, and D) could be identified with substantial similarity across subjects, which explained about 80% of the total data variance (Koenig et al., 1999; Van de Ville et al., 2010). Therefore, following previous research, all instantaneous map topographies at peaks of GFP (Lehmann and Skrandies, 1984) were clustered into four classical map topographies using modified K-means clustering algorithm (Pascualmarqui et al., 1995) for the LOL experts and non-experts, respectively. The polarity of map topographies was neglected during these computations.

The analysis included the following steps (Gao et al., 2017). For each participant, all original maps were clustered into four classes of prototype maps by the global map dissimilarity (GMD) (Van de Ville et al., 2010; Gao et al., 2017). Specifically, four maps – randomly selected from all original maps – were used as the initial prototype maps. The GMD between each prototype map and all original maps were calculated, and each original map was marked as one of the prototype maps with the lowest GMD value. All original maps with the same marker were then averaged, and the obtained maps were used as new prototype maps. The global explained variance (GEV), defined as the sum of squared spatial correlations between each original map and the new prototype map with the same label and weighted by the standard deviation of the electrode values at each point, was computed (Pascualmarqui et al., 1995). Next, the original maps were fitted to new prototype maps using lowest GMD values and were remarked. New averaged maps and GEV were obtained again. This remarking procedure was repeated 100 times until the GEV reached to a maximal value. Thus, the GEV value of the final prototype maps was maximal. Given the initial prototype maps were randomly selected and may affect the qualities of map segmentation, new initial prototype maps were randomly repeatedly selected and the entire above procedure was repeated. Finally, the results of the maximal GEV were used. The group-level prototype maps of four EEG microstates (i.e., class A, B, C and D) were calculated from individual prototype maps, using a procedure described above. Each original map of each participant was assigned to one of the four EEG microstates using minimal GMD between the tested original map and the four group-level prototype maps as a criterion.

*Microstate topographies.* To test the global difference between each two map topographies among four microstates for each group, a topographic analysis of variance (TANOVA) method was used in RAGU (Koenig et al., 2011). The TANOVA method calculated the GMD (Lehmann and Skrandies, 1984) between each two map topographies and established the probability of the observed differences using topographical randomization statistics (random times were 5000).

*Mean duration, Occurrence, and Coverage*. Three parameters of the four microstate classes were computed for each participant: Mean Duration (the average time in milliseconds all covered by a same microstate class), Occurrence (mean number of occurrences of a given microstate class per second), and Coverage (percentage of time coverage of a given microstate class). A two-way ANOVA were conducted with a between-subject factor (Group: experts and non-experts) and a within-subject factor (Microstate: microstate A, B, C, and D) for three microstate parameters, respectively. Significant interactions involving Group were further analyzed through *post hoc* independent samples *t*-tests.

*Microstate syntax*. The microstate syntax was measured as the concatenation between microstate classes over time (Lehmann et al., 2005; Schlegel et al., 2012). The amount of microstate transition from a given class to another class was obtained for each participant; these figures were normalized to fractions of all between-class transitions of the subject, as called transition probabilities. Thus, 12 possibilities were estimated, as there were six possible class pairs (AB, AC, AD, BC, BD, and CD) and each class pair had two possible directions of microstate transition. To test whether the observed transition values were proportional to the relative occurrence of the microstate classes, we calculated the expected probability of transition based on occurrence (Lehmann et al., 2005). The observed and expected transition probabilities were then averaged across participants for each group. The difference between mean observed and expected transition probabilities was conducted by chi-square distance, and the significance of chi-square distance was calculated by a randomization test according to the method used in past research (Lehmann et al., 2005). The tests obtained *p* values of 0.007 and 0.003 for the LOL non-experts and experts, respectively, suggesting that observed transition probabilities included a structure that cannot be interpreted as the occurrence of the microstates. Thus, two-way ANOVAs (group × transition probability) were conducted, and significant interactions involving Group were further analyzed through *post hoc* independent samples *t*-tests. Then, we examined the directional asymmetries of microstate transitions between 6 microstate pairs (i.e., directional predominance). Specifically, the difference in the transition probability between two corresponding microstate classes was computed ([X→Y] – [Y→X]), which allowed us to determine the directional predominance (X ↔ Y). To test directional predominance between groups, two-way ANOVAs (group × directional predominance) were conducted, and significant interactions involving Group were further analyzed through *post hoc* independent samples *t*-tests.

Research suggests that the temporal characteristics of EEG microstates (i.e., Mean Duration, Occurrence, and Coverage) may reflect the temporal course of ongoing cognitive activities (Gao et al., 2017). Furthermore, transition probabilities between microstates may indicate encoding sequential activation of the neural component that generated EEG microstates (Khanna et al., 2015). Thus, these variables of EEG microstates were closely associated with the temporal complexity of the cognitive processes.

### Omega Complexity Analysis

Based on previous research (Kondakor et al., 1999; Gao et al., 2017, 2018), the omega complexity of each frequency band was computed using the LORETA 2015 software. The global omega complexity (all scalp electrodes) and the regional omega complexity (anterior: FP1, FP2, FT9, FT10, F3, F4, F7, and F8; posterior: P7, P3, PZ, P4, P8, O1, OZ, and O2) were computed, respectively. The omega complexity of eight frequency bands was computed: delta (2–4 Hz), theta (4–8 Hz), alpha1 (8–10.5 Hz), alpha2 (10.5–13 Hz), beta1 (13–20 Hz), beta2 (20–30 Hz), gamma1 (30–40 Hz), gamma2 (40–60 Hz). The omega complexity can indicate the rs-EEG spatial complexity through a value ranging between 1 and m (Gao et al., 2017). The lowest value (i.e., 1) means that the data contain only one principal component or spatial mode, indicating a maximum level of synchronization of EEG signals across different scalp locations. The maximum value (i.e., m) means that the total data variance is uniformly distributed across all m principal components, indicating a maximum level of spatial complexity (Kondakor et al., 2005; Gao et al., 2017). The upper bound on the maximum value is equal to the number of electrodes involved in the calculation. Then, for each frequency band, independent samples *t*-tests were performed to compare the LOL experts and non-experts at global, anterior and posterior regions, respectively. A Cohen’s *d* was computed for an independent samples *t*-test to index the effect size if a significant result emerged. For all analyses, false discovery rate (FDR) adjusted *p*-values were used for multiple comparisons (Benjamini and Hochberg, 1995) (*p* < 0.05 was considered statistically significant).

### Results and Discussion

#### Microstate Topographies (see Figure 1A)

The map topographies of microstate classes A, B, C, and D were consistent to previous findings (Koenig et al., 1999). The four microstate classes accounted for 78.06 (S.D. = 3.17%) and 76.22 (S.D. = 4.70%) of the data variance in the LOL non-experts and experts, respectively. For both the LOL experts and non-experts, TANOVA results showed that the map topographies of the four microstate classes significantly differed from each other (*p’s* < 0.001, explained variances [EVs] > 41.68%).

**FIGURE 1:**
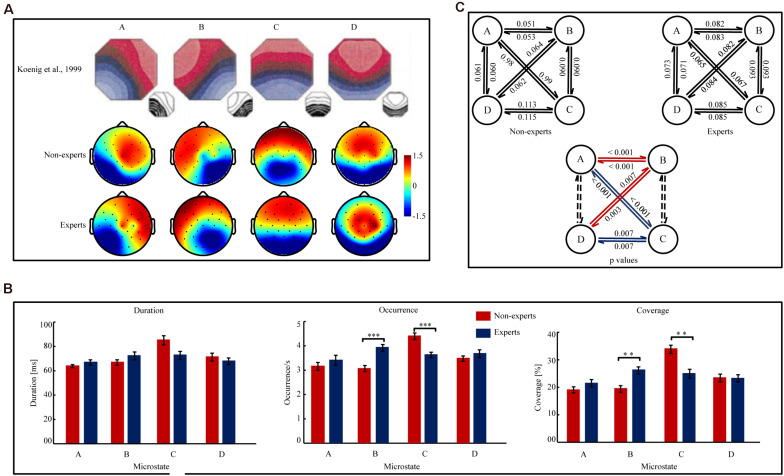
The map topographies of microstate A, B, C, and D and the variables of the temporal complexity in the League of Legends (LOL) non-experts and experts. **(A)** The map topographies of microstate A, B, C, and D of the control group reported in Koenig et al. (1999) and the LOL non-experts and experts in the current study. Microstate A exhibits a left-right orientation, microstate B exhibits a right-left orientation, microstate C exhibits an anterior-posterior orientation, and microstate D exhibits a fronto-central maximum. The color bar of topographies ranges from –1.5 μV to 1.5 μv and the values indicate the average potential of global field potential (GFP). Red color indicates positive values and blue color indicates negative values (can be inverted). **(B)** The Mean duration, Occurrence, and Coverage of microstate A, B, C, and D in the LOL non-experts and experts. Error bars represent SEM ^∗∗^*p*_[FDR]_ < 0.01, ^∗∗∗^*p*_[FDR]_ < 0.001. **(C)** Figure 1C (the two figures in upper part of Figure 1C) indicated the transition probabilities from one microstate to other microstate for all four microstates in the LOL non-experts and experts. The arrow indicated the transition direction from a given microstate to other one. The value on the arrow indicated the transition probabilities. Figure 1C (the one figure in the *lower* part of Figure 1C) indicated the difference of the transition probabilities between the LOL non-experts and experts. The value on the arrow indicated the significant *p*_[FDR]_ values of the transition probabilities between the LOL non-experts and experts. The red arrows indicated that the LOL experts had *higher* transition probabilities than the non-experts, while the blue arrows indicated that the LOL experts had lower transition probabilities than the non-experts. The dashed lines indicated transition probability did not significantly differ between groups.

#### Mean Duration, Occurrence, and Coverage (see Figure 1B)

Three two-way ANOVAs (group × microstate) analyses did not show a significant main effect of group (*p’*s > 0.28). However, a significant group × microstate interaction emerged in the analyses of Mean Duration [*F*(3,144) = 4.07, *p* = 0.01, η*^2^_*p*_* = 0.08], Occurrence [*F*(3,44) = 12.09, *p* < 0.001, η*^2^_*p*_* = 0.20], and Coverage [*F* (3,144) = 8.54, *p* < 0.001, η*^2^_*p*_* = 0.15], respectively. Then, separate independent samples *t*-tests analyzed the between-group differences for each microstate in Mean Duration, Occurrence, and Coverage, respectively. The analyses on microstate B showed that Occurrence [*t*(48) = 4.50, *p*_[FDR]_ < 0.001, Cohen’ *d* = 1.28] and Coverage [*t*(48) = 3.67, *p*_[FDR]_ = 0.001, Cohen’ *d* = 1.05] were significantly *higher* in the experts than in the non-experts, but Mean Duration did not significantly differ between groups (*p*_[FDR]_ = 0.31). The analyses on microstate C showed that Occurrence [*t*(48) = 4.09, *p* < 0.001, Cohen’ *d* = 1.16)] and Coverage [*t*(48) = 3.84, *p* = 0.001, Cohen’ *d* = 1.09] were significantly *lower* in the experts than in the non-experts, but the Mean Duration did not significantly differ between groups (*p*_[FDR]_ = 0.10). The analyses on microstate A and D showed no significant between-group differences (*p*_[FDR]_*’s* > 0.20).

#### Microstate Syntax (see Figure 1C)

A two-way ANOVA (group × transition probability) showed that the main effect of group was not significant (*p* = 0.27), but the group × transition probability interaction was significant [*F*(11,528) = 10.33, *p* < 0.001, η*^2^_*p*_* = 0.18]. Then, 12 separate independent sample *t*-tests were conducted to compare the difference of the transition probabilities for each microstate transition pair, since as there are six possible class pairs (AB, AC, AD, BC, BD, CD) and each class pair has two possible directions of microstate transition. The results showed that the transition probabilities between microstate A and B (A→B: *t*(48) = 5.01, *p*_[FDR]_ < 0.001, Cohen’ *d* = 1.43; B→A: *t*(48) = 5.06, *p*_[FDR]_ < 0.001, Cohen’ *d* = 1.44) and those between microstate B and D (B→D: *t*(48) = 3.43, *p*_[FDR]_ = 0.003, Cohen’ *d* = 0.98; D→B: *t*(48) = 2.98, *p*_[FDR]_ = 0.007, Cohen’ *d* = 0.85) were significantly *higher* in the LOL experts than in the non-experts. However, the transition probabilities between microstate A and C (A→C: *t*(48) = 5.02, *p*_[FDR]_ < 0.001, Cohen’ *d* = 1.43; C→A: *t*(48) = 4.97, *p*_[FDR]_ < 0.001, Cohen’ *d* = 1.42) and those between microstate C and D (C→D: *t*(48) = 3.05, *p*_[FDR]_ = 0.007, Cohen’ *d* = 0.87; D→C: *t* [48] = 3.01, *p*_[FDR]_ = 0.007, Cohen’ *d* = 0.86) were significantly *lower* in the LOL experts than in the non-experts. However, the transition probabilities between microstate A and D and those between microstate B and C did not significantly differ between groups (*p*_[FDR]_’s > 0.08). Additionally, the directional predominance did not significantly differ between groups for microstate A, B, C, or D (*p*’s > 0.05).

#### Omega Complexity (see Figure 2)

The results of the global and anterior omega complexity did not reveal significant differences between the LOL experts and non-experts for any frequency band (*p’s* > 0.05). However, the posterior omega complexities were marginally *higher* in the LOL experts than the non-experts for gamma2 (*t* [48] = 2.17, *p* = 0.04, Cohen’ *d* = 0.62). But this difference between two groups did not withstand FDR correction (*p*_[FDR]_ = 0.22).

**FIGURE 2:**
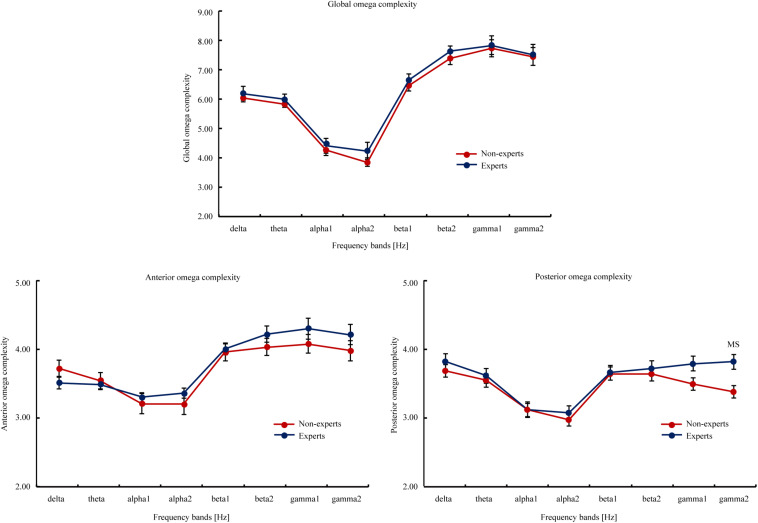
The global, anterior, and posterior omega complexity of the eight EEG frequency bands (i.e., delta, theta, alpha1, alpha2, beta1, beta2, gamma1, and gamma2) in the LOL non-experts and experts. The global omega complexity involved all scalp electrodes; the anterior omega complexity involved FP1, FP2, FT9, FT10, F3, F4, F7, and F8; the posterior omega complexity involved P7, P3, PZ, P4, P8, O1, OZ, and O2. The eight frequency bands involved delta (2–4 Hz), theta (4–8 Hz), alpha1 (8–10.5 Hz), alpha2 (10.5–13 Hz), beta1 (13–20 Hz), beta2 (20–30 Hz), gamma1 (30–40 Hz), and gamma2 (40–60 Hz). Attain values from the interval 1 to m, the lowest value (i.e., 1) means the data is consisted of exactly one principal component or spatial mode, and a maximum synchronization between EEG signals at different scalp locations. The highest value (i.e., m) indicates the total data variance is uniformly distributed across all m principal component, and a maximum spatial complexity is observed. The upper bound on the maximum value is equal to the number of electrodes involved in the calculation. Error bars represent Mean ± SEM. MS indicated marginal significance after FDR correction.

Thus, Experiment 1 showed significant differences in rs-EEG temporal complexity and a tendency of difference in rs-EEG spatial complexity between the LOL experts and non-experts. The findings demonstrated that the LOL experts and non-experts had different brain electrical activities even in absence of tasks and instructions, thus suggesting that ARSG experience is related to certain cognitive improvements. However, it is unclear whether this effect is due to an AVG experience in general or is specific to the ARSG used in this Experiment 1. To examine the generalizability of the findings of Experiment 1, we should also examine other new AVG subgenre other than ARSG.

## Experiment 2

Experiment 2 examined the relationship between PUBG experience and rs-EEG temporal and spatial complexity by comparing the rs-EEG microstate and omega complexity between PUBG experts and non-experts. PUBG game is a multi-player online shooting games (MOSG), where up to one hundred players parachute onto an island and scavenge for weapons and equipment to defeat others while avoiding getting defeated themselves. As the game proceeds, the available safe area of the battleground map decreases in size, leading surviving players into tighter areas to force encounters. PUBG is a “last man standing” game, where the last player or team standing wins the round. PUBG was used in this experiment for three reasons. First, PUBG and LOL are different subgenres of AVG. Yet, they both are popular online, organized, multiplayer competitions requiring not only sensorimotor skills but also strategizing and cooperating with teammates. Thus, the use of PUBG allows us to determine whether the findings of Experiment 1 was merely specific to ARSG. Second, there is substantial evidence supporting the effect of first or third person shooting games experience on the development of various cognitive abilities (Bediou et al., 2018; Bavelier and Green, 2019) such as attention (Green and Bavelier, 2003), visuospatial processing (Green and Bavelier, 2006), working memory and vision (Blacker et al., 2014). Third, to our knowledge, little research on video gaming-related cognitive improvement has used PUBG. Yet, research has not yet examined the relationship between PUBG experience and the rs-EEG temporal and spatial complexity. Thus, this experiment can contribute to the literature by investigating the relationship between temporal and spatial complexity and the experience of playing a highly popular but little researched MOSG genre.

### Participants

Experiment 2 followed the recruitment procedure and requirements used in Experiment 1, except that the participant in Experiment 2 reported their PUBG gaming experience and expertise, their PUBG ID, and their video gaming experience of genres other than PUBG in the immediate recent 2 years, which was used to ensure that PUBG was the primary game genre for all participants, including both experts and non-experts, recruited in this experiment. Five additional individuals were excluded from the final sample, because they had moderate/severe depression or anxiety (i.e., SDS ≥ 63 or SAS ≥ 60) (*n* = 3), or Internet gaming disorders (*n* = 2). All participants were right-handed and had a normal or corrected-to-normal vision and no history of mental and neurological diseases. The participants were 22 PUBG experts (*M* = 20.41 years, *SD* = 1.71; male = 20) and 27 PUBG non-experts (*M* = 20.37 years, *SD* = 1.90; male = 26). The experts had at least 2 years of PUBG gaming experience and were PUBG masters based on their Expertise Gaming Ranking provided by the PUBG gaming software (the top 7% of players). The non-experts had less than 0.5 years of PUBG gaming experience and were non-experts according to their Expertise Gaming Rankings (the lowest 29.92-45.11% of players).

### Procedure

Experiment 2 followed the procedures of EEG recording and preprocessing, EEG microstate analysis, and omega complexity analysis used in Experiment 1. On an average, 143.93 (*SD* = 21.55) artifact-free EEG epochs were available for each expert, and 140.32 (*SD* = 16.01) epochs for each non-expert. The number of available epochs did not significantly differ between groups (*p* = 0.65). In addition, the randomization tests of the transition probability obtained *p* values of 0.006 and 0.004 for the PUBG non-experts and the experts, respectively, suggesting that observed transition values included a structure that cannot be interpreted as the occurrence of the microstates.

### Results and Discussion

#### Microstate Topographies (see Figure 3A)

The map topographies of microstate classes A, B, C, and D were consistent to previous findings (Koenig et al., 1999). The four microstate classes accounted for 78.82 ± 3.6% of the data variance across the PUBG non-experts, and 75.08 ± 4.0% of the data variance across experts. For both the PUBG experts and non-experts, TANOVA results showed that the map topographies of all four microstate classes were significantly different from each other (*p’s* < 0.001, EVs > 39.84%).

**FIGURE 3:**
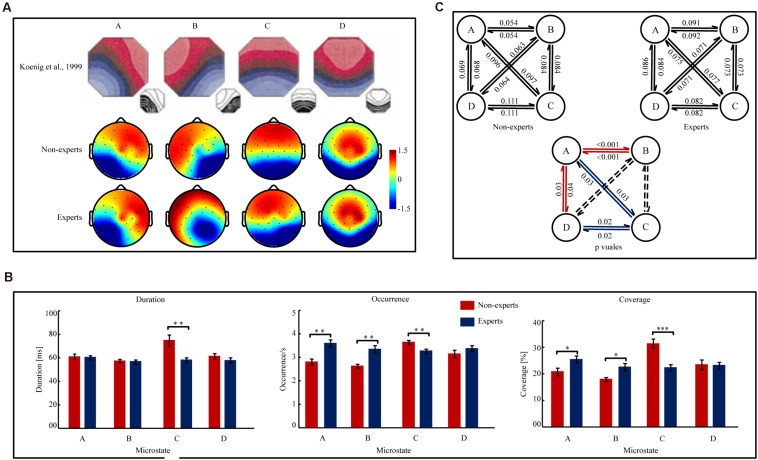
The map topographies of microstate A, B, C, and D and the parameters of the temporal complexity in Player Unknown’s Battle Grounds (PUBG) non-experts and experts. **(A)** The map topographies of microstate A, B, C, and D of control group reported in Koenig et al. (1999) and PUBG non-experts and experts. Microstate A exhibits a left-right orientation, microstate B exhibits a right-left orientation, microstate C exhibits an anterior-posterior orientation, and microstate D exhibits a fronto-central maximum. The color bar of topographies ranges from –1.5 μV to 1.5 μv and the values indicate the average potential of global field potential (GFP). Red color indicates positive values and blue color indicates negative values (can be inverted). **(B)** The Mean duration, Occurrence, and Coverage of microstate A, B, C, and D in PUBG non-experts and experts. Error bars represent Mean ± S.E.M. ^∗^*p*_[FDR]_ < 0.05, ^∗∗^*p*_[FDR]_ < 0.01, ^∗∗∗^*p*_[FDR]_ < 0.001. **(C)** Figure 1C (the two figures in upper part of Figure 1C) indicated the transition probabilities from one microstate to other microstate for all four microstates in PUBG non-experts and experts. The arrow indicated the transition direction from a given microstate to other one. The value on the arrow indicated the transition probabilities. Figure 1C (the one figure in the *lower* part of Figure 1C) indicated the difference of the transition probabilities between PUBG non-experts and experts. The value on the arrow indicated the significant *p*_[FDR]_ values of the transition probabilities between PUBG non-experts and experts. The red arrow indicated PUBG experts had *higher* transition probabilities than non-experts. While the blue arrow indicated PUBG experts had *lower* transition probabilities than non-experts. The dashed lines indicated transition probability did not significantly differ was not significant between groups.

#### Mean Duration, Occurrence, and Coverage (see Figure 3B)

Three separate two-way ANOVAs (group × microstate) analyzed the three variables, respectively. The analysis on Mean Duration showed a main effect of group [*F*(1,47) = 8.76, *p* = 0.005, η*^2^_*p*_* = 0.16] and a significant group × microstate interaction [*F*(3,141) = 4.45, *p* = 0.01, η*^2^_*p*_* = 0.09]. The analysis on Occurrence also revealed a main effect of group [*F*(1,47) = 13.25, *p* < 0.001, η*^2^_*p*_* = 0.22] and a group × microstate interaction [*F*(3,141) = 9.26, *p* < 0.001, η*^2^_*p*_* = 0. 17]. However, the analysis on Coverage showed that the main effect of group was not significant (*p* = 0.19), but the group × microstate interaction was significant [*F*(3,141) = 7.52, *p* < 0.001, η*^2^_*p*_* = 0.14]. Then, separate independent samples *t*-tests analyzed the between-group differences for each microstate in Mean Duration, Occurrence, and Coverage, respectively. The analyses on microstate A showed that the PUBG experts had a significantly *higher* Occurrence [*t*(47) = 4.10, *p*_[FDR]_ < 0.001, Cohen’ *d* = 1.18] and Coverage [*t*(47) = 2.41, *p*_[FDR]_ = 0.03, Cohen’ *d* = 0.69] than the non-experts, but the Mean Duration did not significantly differ between groups (*p*_[FDR]_ = 0.79). The analyses on microstate B showed that the PUBG experts had a significantly *higher* Occurrence [*t*(47) = 3.93, *p*_[FDR]_ < 0.001, Cohen’ *d* = 1.13] and Coverage [*t*(47) = 2.94, *p*_[FDR]_ = 0.01, Cohen’ *d* = 0.84] than the non-experts, but the Mean Duration did not significantly differ between groups (*p*_[FDR]_ = 0.77). The analyses on microstate C showed that the PUBG experts had a significantly *lower* Mean Duration [*t*(47) = 3.34, *p*_[FDR]_ = 0.008, Cohen’ *d* = 0.96], Occurrence [*t*(47) = 2.93, *p*_[FDR]_ = 0.007, Cohen’ *d* = 0.84], and Coverage [*t*(47) = 4.22, *p*_[FDR]_ < 0.001, Cohen’ *d* = 1.21] than the non-experts. The analyses on microstate D showed no significant between-group differences (*p*_[FDR]_*’s* > 0.29).

#### Microstate Syntax (see Figure 3C)

A two-way ANOVA (group × transition probability) revealed a significant main effect of group [*F*(1, 47) = 10.80, *p* = 0.002, η*^2^_*p*_* = 0.18] and a significant group × microstate transition interaction [*F*(11,517) = 8.66, *p* < 0.001, η*^2^_*p*_* = 0.16]. Then, 12 separate independent sample *t* tests were conducted to compare the difference of the transition probabilities for each microstate transition pair. The results showed that the transition probabilities between microstate A and B (A→B: *t*(47) = 5.05, *p*_[FDR]_ < 0.001, Cohen’ *d* = 1.45; B→A: *t*(47) = 5.05, *p*_[FDR]_ < 0.001, Cohen’ *d* = 1.45) and those between microstate A and D (A→D: *t*(47) = 2.65, *p*_[FDR]_ = 0.02, Cohen’ *d* = 0.76; D→A: *t*(47) = 2.67, *p*_[FDR]_ = 0.02, Cohen’ *d* = 0.77) were significantly *higher* in the PUBG experts than in the non-experts. However, the transition probabilities between microstate A and C (A→C: *t*(47) = 2.36, *p*_[FDR]_ = 0.03, Cohen’ *d* = 0.68; C→A: *t*(47) = 2.51, *p*_[FDR]_ = 0.03, Cohen’ *d* = 0.72) and those between microstate C and D (C→D: *t*(47) = 2.91, *p*_[FDR]_ = 0.02, Cohen’ *d* = 0.84; D→C: *t*(47) = 2.96, *p*_[FDR]_ = 0.02, Cohen’ *d* = 0.85) were significantly *lower* in the PUBG experts than in the non-experts. However, the analyses did not show significant findings for the transition probabilities of the other microstate pairs (*p*_[FDR]_*’*s > 0.10). Additionally, the directional predominance did not significantly differ between groups for microstate class A, B, C, or D (*p’s* > 0.25).

#### Omega Complexity (see Figure 4)

The results showed that compared to the PUBG non-experts, the experts had significantly *higher* global omega complexity in alpha1, alpha2, beta1 (*t*_[max]_ = 2.72, *p*_[min]_ = 0.01). However, the between-group difference in beta1 did not withstand the FDR correction (*p*_[FDR]_ = 0.07). The PUBG experts showed *higher* posterior omega complexity than the non-experts in beta2 [*t*(47) = 2.07, *p* = 0.04, Cohen’ *d* = 0.60]; but this difference did not withstand the FDR correction (*p*_[FDR]_ = 0.32).

**FIGURE 4:**
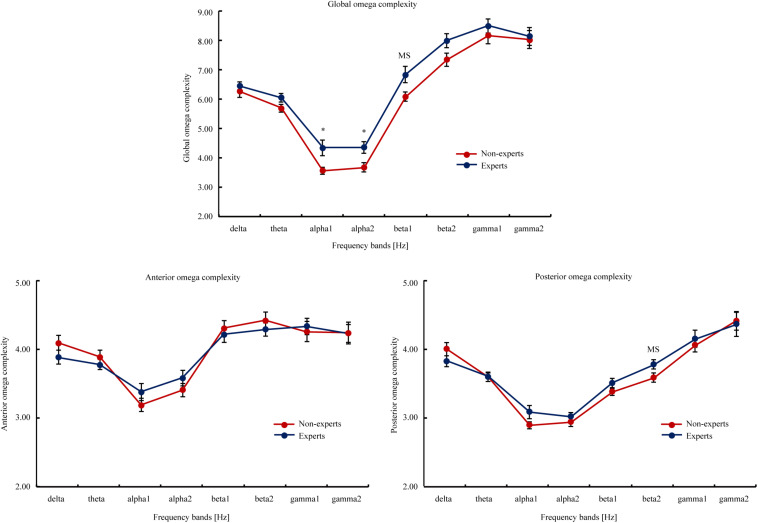
The global, anterior, and posterior omega complexity of the eight EEG frequency bands (i.e., delta, theta, alpha1, alpha2, beta1, beta2, gamma1, and gamma2) in the PUBG non-experts and experts. The global omega complexity involved all scalp electrodes; the anterior omega complexity involved FP1, FP2, FT9, FT10, F3, F4, F7, and F8; the posterior omega complexity involved P7, P3, PZ, P4, P8, O1, OZ, and O2. The eight frequency bands involved delta (2–4 Hz), theta (4–8 Hz), alpha1 (8–10.5 Hz), alpha2 (10.5–13 Hz), beta1 (13–20 Hz), beta2 (20–30 Hz), gamma1 (30–40 Hz), and gamma2 (40–60 Hz). Attain values from the interval 1 to m, the lowest value (i.e., 1) means the data is consisted of exactly one principal component or spatial mode, and a maximum synchronization between EEG signals at different scalp locations. The highest value (i.e., m) indicates the total data variance is uniformly distributed across all m principal component, and a maximum spatial complexity is observed. The upper bound on the maximum value is equal to the number of electrodes involved in the calculation. Error bars represent mean ± S.E.M. MS indicated marginal significance after FDR correction, * *p*_[FDR]_ < 0.05.

Thus, Experiment 2 showed significant differences in rs-EEG temporal and spatial complexity between the PUBG experts and non-experts. The findings support that the generalizability of the findings of Experiment 1.

## General Discussion

This study examined the relationship between ARSG/MOSG experience and cognitive improvements by examining the rs-EEG microstate and omega complexity in LOL/PUBG experts and non-experts. Results showed significant differences in rs-EEG microstate and omega complexity between LOL/PUBG experts and non-experts, suggesting that AVG playing experience is related to the rs-EEG temporal and spatial complexity.

### The rs-EEG Temporal and Spatial Complexity Related to LOL Experience

Experiment 1 found that the Occurrence and Coverage of microstate B were *higher* in the LOL experts than the non-experts. Microstates temporal parameters speak to large-scale neural assemblies. In specific, duration reflects the stability of each microstate and is influenced by cortical and subcortical activity. Occurrence indicates the tendency of certain neural generators activated within a given time period. Coverage reveals the amount of total recording time that neural generators remain dominant (Milz et al., 2016). Since the temporal dynamics of different microstates reflected the dynamic synchronization of different neural networks, it may indicate distinctive cognitive processes or mental states. The four EEG microstates may be related to the large-scale resting-state fMRI networks (Britz and Michel, 2010; Van de Ville et al., 2010). In specific, microstate B is related to primary sensory processing, such as visual system, spatial attention, and mental imagery (Britz and Michel, 2010; Custo et al., 2017; Seitzman et al., 2017). Thus, long-term LOL experience may be associated with more occurrence tendency and more cognitive processing related to microstate B, such as primary visual processing. This is also consistent with previous findings that AVG experience is related to improved visual cognitive processing (Green and Bavelier, 2007; Li et al., 2015) and increased gray matter volume in the occipital cortex (Kuehn and Gallinat, 2014), because LOL players are exposed to a rapidly changing and visually rich environment in a gaming session (Bavelier and Green, 2019). However, it should be noted that there is also evidence suggesting that microstate A is related to visual processing while microstate B is related to *auditory* processing (Milz et al., 2016). Thus, the functional significance of microstates under an AVG setting still requires further research.

We found that the Occurrence and Coverage of microstate C were *lower* in the LOL experts than the non-experts, suggesting long-term LOL experience is associated with more occurrence tendency and the amount of cognitive processing related to microstate C. This is consistent with past findings that (1) microstate C was related to the *anterior* default mode network (aDMN) – a task-negative network (Koenig et al., 1999; Sestieri et al., 2011; Milz et al., 2016; Seitzman et al., 2017; Michel and Koenig, 2018), and (2) the association between AVG experience and the enhanced DMN is mostly evident in the *posterior* regions (e.g., bilateral precuneus, parahippocampal gyrus, right angular gyrus – (Gong et al., 2019b). Thus, the current findings support the view that the aDMN may be less activated in AVG experts than non-experts. Reduced neural activity in resting-state networks may be related to improved neural efficiency, which may enhance performance on a cognitive task. Perhaps, the cognitive resources of the aDMN is reallocated to the task-related networks in the LOL experts (e.g., the central executive network, the salience network) (Gong et al., 2016), supporting their superior gaming skills. However, microstate C can also be related to the cingulo-opercular system – including the cognitive control network, anterior cingulate, and insula, especially the salience network (Seeley et al., 2007; Britz and Michel, 2010) – which is positively related to task performance and cognitive control (Coste and Kleinschmidt, 2016). Based on these findings, one may predict that LOL experts should have increased microstate C compared to non-experts. However, the current findings do not support this prediction.

The transitions between microstates may indicate the sequential activation of the different networks, and the time sequence of the microstates may represent the switching between the activation of the underlying neural assemblies (Khanna et al., 2015). Therefore, the *higher* transition between A and B in the LOL experts compared to the non-experts may reflect a more sufficient switching between the auditory and visual networks in the experts. Similarly, the current findings suggest that compared to the LOL non-experts, the experts have a more sufficient switching between the visual and dorsal attention networks (microstate D) that reflected focus switching and reflexive aspects of attention (Britz and Michel, 2010). Furthermore, the switching between the auditory and aDMN networks and that between the aDMN and dorsal attention networks are less sufficient in the experts than in the non-experts. Thus, the current findings suggest that long-term LOL experience is related to the altered temporal complexity of EEG signals.

The posterior omega complexity was marginally *higher* in gamma2 in the LOL experts than the non-experts, suggesting that the experts had more independent and parallel processing than the non-experts in posterior regions (Gao et al., 2017). Thus, the spatial complexity may be *higher* in the experts than the non-experts. High spatial complexity may indicate that new regions of a complex multidimensional state space are developing in the posterior region, which improves its self-replication and persistence and therefore opens new possibilities of life (Jeffery and Rovelli, 2020). However, this should be interpreted with caution because the omega complexity did not differ between the LOL experts and the non-experts when the FDR correction was applied. In addition, gamma-band signals may be influenced by muscle artifacts. Post beta 2, there is also a tendency for the complexity values to increase. Furthermore, one’s AVG duration or experience may be related to their ability to sit still.

### Relevant rs-EEG Temporal and Spatial Complexity in PUBG Experience

To examine the generalizability of the findings of Experiment 1, Experiment 2 examined the relationship between PUBG experience and rs-EEG temporal and spatial complexity. Compared to the PUBG non-experts, the experts had *higher* Occurrence and Coverage for microstate B but *lower* Occurrence and Coverage for microstate C – a pattern of results consistent to Experiment 1. Additionally, the PUBG experts had *lower* duration than the non-experts, suggesting that the experts may have faster switches in microstate C and thereby a higher rate of information processing (Brodbeck et al., 2012). In addition, compared to the PUBG non-experts, the experts had *higher* Occurrence and Coverage of microstate A, which is associative with the auditory network critical for verbal/phonological processing or sensorimotor system (Britz and Michel, 2010). This should be related to the fact that PUBG requires players to locate the source of gunshot sounds and to vocally and verbally communicate with their teammates. Indeed, AVG/video game experience has been found related to improved auditory cognition (Donohue et al., 2010; Green et al., 2010), reading speeds, and literacy (Franceschini et al., 2017).

The results of the transition probabilities suggested that compared with the PUBG non-experts, the experts had a more sufficient switching between the auditory and visual networks, but a less sufficient switching between the auditory and aDMN networks and between the aDMN and dorsal attention networks (Britz and Michel, 2010; Khanna et al., 2015). In addition, the PUBG experts had *higher* transition between the auditory and dorsal attention networks than the non-experts, perhaps because PUBG emphasizes auditory processing. Thus, the current findings suggest that long-term PUBG experience is related to the altered temporal complexity of the EEG large-scale networks.

In addition, the results of omega complexity showed that the PUBG experts had more independent and parallel processing than the non-experts in global brain regions and posterior regions (Gao et al., 2017), suggesting that spatial complexity may be higher in the experts than the non-experts.

### Distinct rs-EEG Temporal and Spatial Complexity: Microstate A and Global Complexity

A similar pattern of results was found in Experiments 1 and 2. First, both experiments found significant differences between the experts and the non-experts in Coverage and Occurrence of microstate B and C. This cross-subgenre similarity should be related to the fact that LOL and PUBG share certain common characteristics that tax cognitive processes related to microstates B and C. Indeed, both LOL and PUBG are cognitively demanding tasks just like other AVGs that require players to suppress aDMN activity in a gaming session (Bavelier and Green, 2019). In addition, both LOL and PUBG are rich in visual stimuli, thus placing a heavy demand on visual cognition (Bavelier and Green, 2019). Second, both experiments showed that the LOL/PUBG experts had *higher* transition between microstate A and B and *lower* transition related to microstate C than the non-experts. Third, both experiments revealed a potential between-group difference in omega complexity of the posterior region, although this finding requires further verification.

We also found certain subgenre-specific complexity changes between the experts and non-experts. For example, the PUBG experts had *higher* Occurrence and Coverage of microstate A than non-experts, but this between-group difference was not observed in the LOL experts and non-experts. This may be related to the fact that LOL playing relies primarily on visual information processing, while auditory information only serves as the background soundscape enriching the gaming experience. By contrast, PUBG playing relies on both visual and auditory information processing. PUBG players need to locate the source of the gunshot sounds to further identify the target location. Results of transition also showed that the PUBG experts had a *higher* transition between microstates A and D than non-experts, while the LOL experts had a *higher* transition between microstates B and D. In addition, we found that global omega complexity differed between the PUBG experts and non-expert, but this between-group difference was not observed in the LOL experts and non-experts. However, this finding still requires further verification.

This study had several limitations. First, although neural generators of the EEG microstates have been found related to large-scale resting-state fMRI networks, this relationship still requires further research. Some researchers have hypothesized that the microstates and resting-state networks are one-to-one mappings (Britz and Michel, 2010), while others have proposed that the neural generators of microstates may overlap with each other, and that resting-state networks can be divided into sub-networks for distinct functions (Milz et al., 2017). Thus, the functional significance of the EEG microstates still remains unclear, and the relationship between EEG microstates and fMRI resting-state networks should be interpreted with caution. Second, this study clustered EEG signals to four widely reported canonical resting-state EEG microstate classes based on previous studies (Koenig et al., 1999; Van de Ville et al., 2010). Future research should also use a data-driven clustering algorithm to choose the optimal number of clusters, such as Krzanowski-Lai (KL) criterion (Murray et al., 2008), in order to verify the current findings using an alternative method. Third, the participants in this study were predominately male due to the lack of female gamers in general. Future research should include more female participants to probe the potential gender difference. Fourth, the present study’s correlational nature precludes drawing causal conclusions. Furthermore, it is also possible that there were pre-existing systematic differences between the experts and non-experts. Thus, certain groups of people were more likely to form and maintain AVG playing habits than others. It is therefore unclear whether the between-group differences observed in this study was due to the pre-existing differences or/and the AVG experience. To address this issue, a longitudinal, interventional study should be performed, where participants are randomly assigned to receive (or not to receive) AVG training.

Nevertheless, this study found a clear association between AVG experience and the altered temporal and spatial complexity of the EEG large-scale networks. This association is observable across two AVG subgenres. Furthermore, we found certain subgenre-specific complexity changes. For example, PUBG experience was related to *higher* occurrence and coverage of the auditory processing (microstate A) and higher transition probabilities between the auditory and dorsal attention networks. These findings support the recent proposal that AVG should be categorized based on the gaming mechanics of a specific game rather than a generic genre designation.

## Data Availability Statement

The raw data supporting the conclusions of this article will be made available by the authors, without undue reservation.

## Ethics Statement

This study was approved by the Ethics Review Committee, University of Electronic Science and Technology of China (UESTC), Chengdu, China. The experimental procedures were conducted according to the seventh revision of the Declaration of Helsinki. Informed consents were obtained from all participants in this study.

## Author Contributions

RC, DG, and DY conceived and designed the study and wrote the manuscript. GY and WM contributed to the study design, result interpretation, and first manuscript writing. LZ, JJ, and LD conducted data analysis and result interpretation. ZX and LJ completed data collection. All authors contributed to manuscript revision and agreed to be accountable for all aspects of this study.

## Conflict of Interest

The authors declare that the research was conducted in the absence of any commercial or financial relationships that could be construed as a potential conflict of interest.

## Footnotes

1 http://blogs.wsj.com/digits/2014/01/27/player-tally-for-league-of-legends-surges
